# Gray-white matter ratio in pediatric and adult cardiopulmonary arrest studies: a rapid review

**DOI:** 10.1016/j.resplu.2026.101321

**Published:** 2026-04-11

**Authors:** Kie Honjo, Geraldine Goco, Ahmed Al Khalifah, Nicole McKinnon, Barnaby R. Scholefield, Suzanne Laughlin, Anne-Marie Guerguerian

**Affiliations:** a Neuroscience and Mental Health Program, Research Institute, The Hospital for Sick Children, Toronto, ON, Canada; b Department of Critical Care Medicine, The Hospital for Sick Children, University of Toronto, Toronto, ON, Canada; c Department of Diagnostic and Interventional Radiology, Division of Neuroradiology, The Hospital for Sick Children, Medical Imaging Department, University of Toronto, Toronto, ON, Canada

**Keywords:** Hypoxic-ischemic injury, Head CT, Gray-white matter ratio, Cardiac arrest, Outcome, Adults, Pediatrics

## Abstract

**Objectives:**

The gray-white matter ratio is used to estimate tissue edema on head computed tomography scans in cardiac arrest research. This rapid review aimed to determine whether gray-white matter ratio values differed between studies that included children and adults. We hypothesized that brain maturity affects myelination and, consequently, age would alter the range of the gray-white matter ratio.

**Methods:**

We searched PubMed from 2000 to 2025. Studies were selected if they included children or adult patients with cardiac arrest and reported gray-white matter ratio values relative to neurologic outcome groups or to normal controls. We summarized the studies and explored how gray-white matter values trended with age, gray-white matter ratio equations, timing of image acquisition during the post-resuscitation phase, and neurologic outcome groups.

**Results:**

We retrieved five studies in children and 40 in adults. Regions of interest and equations used to measure gray-white matter ratio varied between studies, but most included the basal ganglia. The gray-white matter ratio was generally lower in poor-outcome groups than in good-outcome groups. In children with good outcomes, gray-white matter ratio values were similar to those of adults with poor outcomes. The gray-white matter ratio values were lower in younger children than in older children and adults. We did not find a consistent relationship between gray-white matter ratio values and the elapsed time from cardiac arrest to image acquisition.

**Conclusions:**

This review revealed varying gray-white matter ratios between children and adults in cardiac arrest studies, underscoring the importance of accounting for age when evaluating gray-white matter ratios in pediatric cardiac arrest populations. Gray-white matter ratio values were dependent on the regions of interest included in the equations. Readers should be aware of these sources of variation when comparing studies that use the gray-white matter ratio as a biomarker of hypoxic-ischemic injury in cardiac arrest. Future work is warranted to optimize the use of the gray-white matter ratio in pediatric cardiac arrest research.

## Highlights

• **Question:** Do gray-white matter ratio values in head computed tomography after cardiac arrest differ between pediatric and adult studies?
• **Findings:** Gray-white matter ratio values were lower in younger children compared to older children and adults. Age altered the gray-white matter ratio values.
• **Meaning:** Predicting outcomes in children using gray-white matter ratio cut-offs characterized in adult populations is not advisable. Our review suggests that pediatric patients may be misclassified into poor-outcome groups if adult ranges are used.

## Introduction

Head computed tomography (CT) after cardiac arrest may assist in diagnosing the cause of the precipitating event. Head CT may also serve as a parameter for neurologic prognostication. Loss of gray-white matter differentiation on head CT is known to be one of the imaging features associated with hypoxic ischemic injury.^1^, ^2^, ^3^ Gray-white matter ratio calculated from mean attenuation in Hounsfield Units (HU) in specific gray-white matter regions has been reported to predict neurologic outcomes in adults and children.^3^, ^4^ Brain magnetic resonance imaging (MRI) is a more sensitive modality for detecting ischemic changes, but several barriers limit its broad utilization. For patients after extracorporeal cardiopulmonary resuscitation (ECPR) supported by extracorporeal membrane oxygenation (ECMO), head CT imaging remains the preferred alternative. Some have published that quantifying abnormalities on CT may be a promising neuroimaging biomarker in adults following ECPR to assist in early decision-making.^5^, ^6^, ^7^, ^8^ As the field moves to develop normative and cut-off values for abnormal findings, we set out to characterize what to expect in pediatric and adult CT scans. We hypothesized that GWR in the pediatric population would differ from that in the adult population.^9^ Consequently, we set out to determine whether GWR values reported in pediatric and adult studies differed. The purpose of this rapid review was to summarize the GWR ranges reported in pediatric cardiac arrest studies and contrast these ranges with values reported in adult studies. We aimed to explore other sources of heterogeneity between studies, such as differing regions of interest used for GWR calculations, elapsed time from cardiac arrest to image acquisition, and measures used to classify outcome groups.

## Methods

We used rapid review methodology over systematic or scoping review methodologies to serve the purpose of our literature review. We set out to answer specific questions about the GWR values reported in cardiac arrest studies: we hypothesized that maturation, technique, timing of imaging could alter GWR values reported.^10^, ^11^ The specific objectives were to list the average GWR values reported in pediatric and adult cardiac arrest studies, to report the regions of interest and the equations used in the calculations, and to report the ranges of values reported between favorable and unfavorable outcome groups.

We used PubMed to search the English-language literature from January 2000 to December 2025. The MeSH terms used were the following: “gray matter white matter ratio”, “gray to white matter ratio”, “gray white matter ratio”, “gray-white matter ratio”, “GWR”, “gray-to-white-matter ratio”, “cardiopulmonary arrest”, “cardiac arrest”, “extracorporeal pulmonary resuscitation”, “ECPR”, “head computed tomography”, “head CT”, “brain computed tomography”, “brain CT”, “neurologic outcome”, and “neurological outcome”. Studies were included for final analysis if they met the following inclusion criteria: (1) population: humans, children (<18 years) following cardiac arrest and/or ECPR, or adults (≥18 years) following cardiac arrest and/or ECPR; (2) GWR values measured on head CT and reported as a median or mean in text or graphs; (3) neurologic or survival outcomes, reported by groups. Two reviewers were involved in screening, inclusion and exclusion, and discrepancies were resolved by consensus with an additional reviewer. Among studies published in adults, several studies included overlapping cohorts or registry samples; in this case, we selected the most recent publication or the publication that reported imaging acquisition time. We extracted information on the studied population: age, cardiac arrest setting (in or out of hospital), and CPR and or ECPR; on the published outcomes and classified them into good- and poor-outcome groups, and dichotomized them based on the type of outcome used in each study. We obtained the CT acquisition kilovoltage when available. We found the elapsed time between the cardiac arrest event and the timing of imaging when reported; if not reported, we used the protocol included in each study’s method section (e.g., within 4 h or within 24 h).

GWR may be measured using different regions of interest and different equations to calculate the ratio between gray and white matter regions. We tallied the regions of interest with the following abbreviations: basal ganglia (GWR_bg); single deep nucleus with caudate nucleus (GWR_si(CN)) or with putamen (GWR_si(PT)); cortical gray matter (GWR_cortical); average of two combined regions, GWR_bg and GWR_cortical (GWR_average). If studies reported GWR values with different gray matter regions, we selected a single equation per study and prioritized the reporting in the following order: (1) basal ganglia; (2) caudate nucleus; (3) putamen; or (4) with any GWR reported (Equations in Supplementary material Table 2).

We organized the data for visualization using summary plots to explore trends. We divided the pediatric studies (Fig. 2) and the adult studies (split across Fig. 3, Fig. 4 to fit the page). We reported average GWR as median with interquartile range (IQR) or mean with standard deviation (SD). Study participants were stratified into three groups: controls (when available), good-outcome (good outcome or survived) or poor-outcome (poor outcome, coma, or death). In the pediatric figure, we sorted the studies by region of interest used (basal ganglia or putamen) and by age (from youngest to oldest). In the adult studies figures, we ordered by region of interest and equation used for GWR calculation from top to bottom and by image acquisition time (earlier to later after the cardiac arrest): (a) basal ganglia, caudate nucleus, and GWR combined average similar to GWR_average (GWR_ave-mod)^12^; (b) putamen; and (c) GWR calculated with whole brain regions (GM/WM, GWR_whole)^3^ or using a modified basal ganglia equation that included the thalamus (GWR_bg2).^13^ Graphs were generated in Excel (Microsoft) for Windows 11, Version 16.0.

**Fig. 1:**
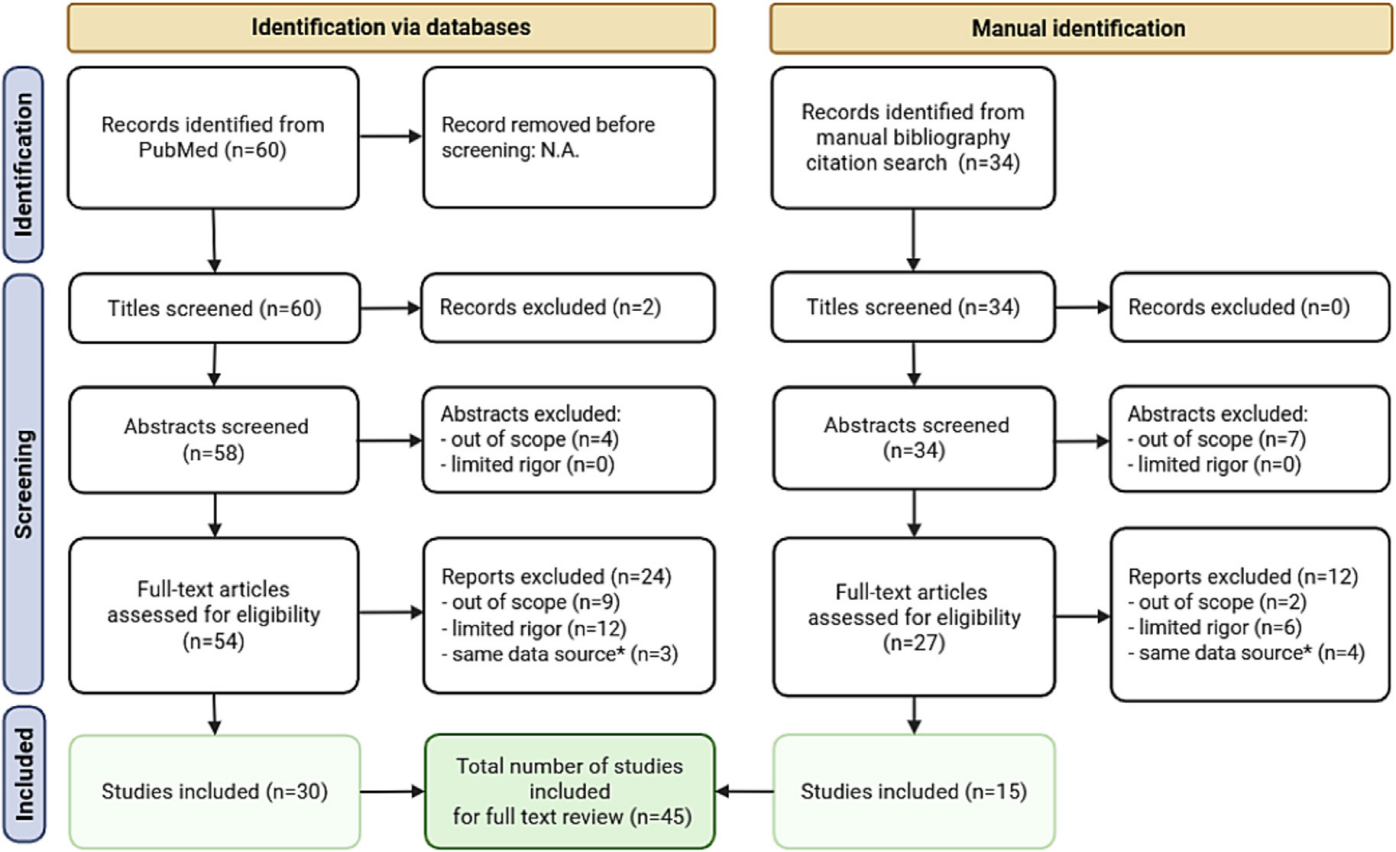
**PRISMA flow diagram**. *“Same data source” is noted where studies reported using a source of data presumed to have been previously included in published analyses based on the institution or cohort’s years of inclusion.

**Fig. 2:**
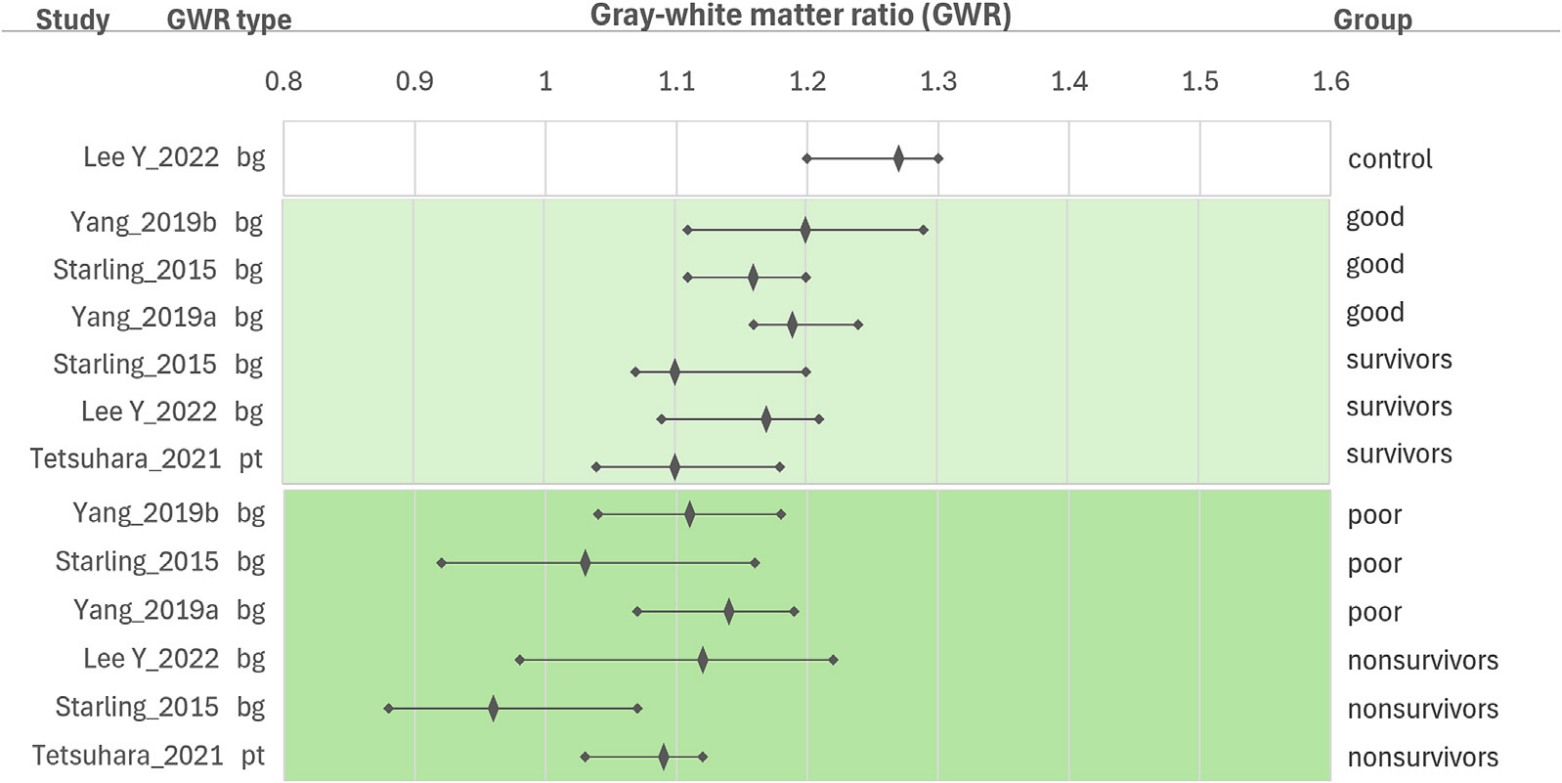
**GWR distribution in pediatric cardiac arrest studies**. The forest plot includes a diamond shape and a line to indicate the median gray-white matter ratio (GWR) with interquartile range or the mean and standard deviation. On the left axis, the first author's last name and publication year indicate a study. The GWR type is shown with ‘bg’ abbreviation referring to the GWR_bg equation calculated with basal ganglia; ‘pt’ abbreviation refers to the GWR_si(PT) equation calculated with putamen. On the right axis, study results are displayed by group: control, good outcome, survivor, poor outcome, and nonsurvivor. Normal controls are shown with a white background, good outcomes or survivors with a light-green color background, and poor outcomes or nonsurvivors with a dark-green color background. (For interpretation of the references to color in this figure legend, the reader is referred to the web version of this article.)

**Fig. 3:**
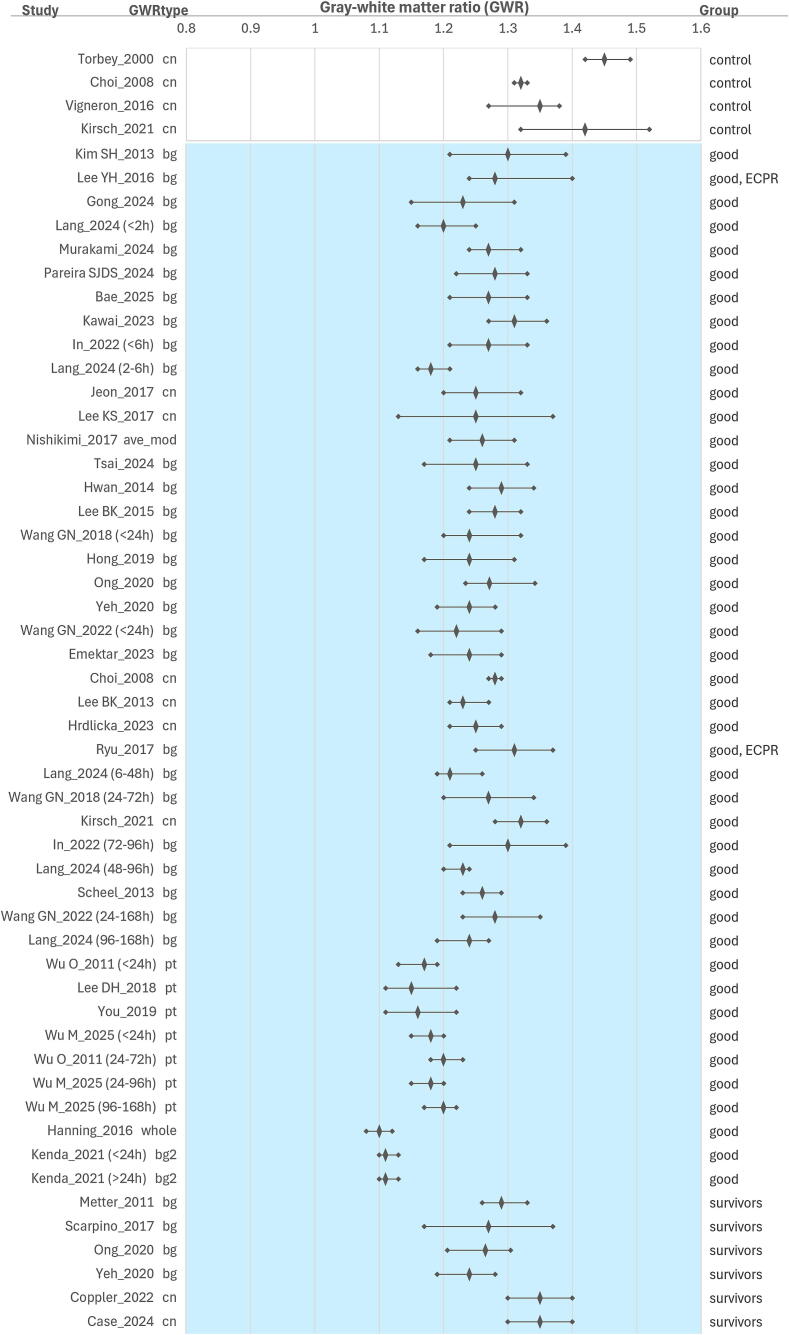
**GWR distribution in adult cardiac arrest and extracorporeal cardiopulmonary resuscitation (ECPR) studies’ favorable groups**. The forest plot includes a diamond shape and a line to indicate the median gray-white matter ratio (GWR) with interquartile range or the mean and standard deviation, for the control, and favorable groups: good outcome or survivors groups. On the left axis, the first author's last name and publication year indicate a study; if studies reported more than one group based on CT acquisition time are indicated in parenthesis for each strata: <2 h, <6 h, 2–6 h, <24 h, >24 h, 6–48 h, 24–72 h, 24–96 h, 72–96 h, 48–96 h, 24–168 h, 96–168 h. The GWR type is shown with the abbreviations ‘cn’, ‘bg’, ‘pt’, ‘ave’, ‘whole’, ‘bg2′, referring to the equations used to calculate the GWR: ‘cn’ with caudate nucleus (GWR_si(CN)), ‘bg’ with basal ganglia (GWR_bg), ‘pt’ with putamen (GWR_si(PT)), ‘ave_mod’ with thalamus and cortical gray matter (GWR_ave_mod), ‘whole’ with whole brain gray mat ter (GWR_whole), ‘bg2′ with basal ganglia plus thalamus (GWR_bg2). Equations are included in Table 2 in the Supplemental Materials. On the right axis, study results are displayed by group: control, good outcome, survivors; groups including ECPR are noted. Normal controls are shown with a white background, good outcomes or survivors with a light-blue color background. Studies are ordered from top to bottom, from early to later CT acquisition time, in each good outcome and survivor group. (For interpretation of the references to color in this figure legend, the reader is referred to the web version of this article.)

**Fig. 4:**
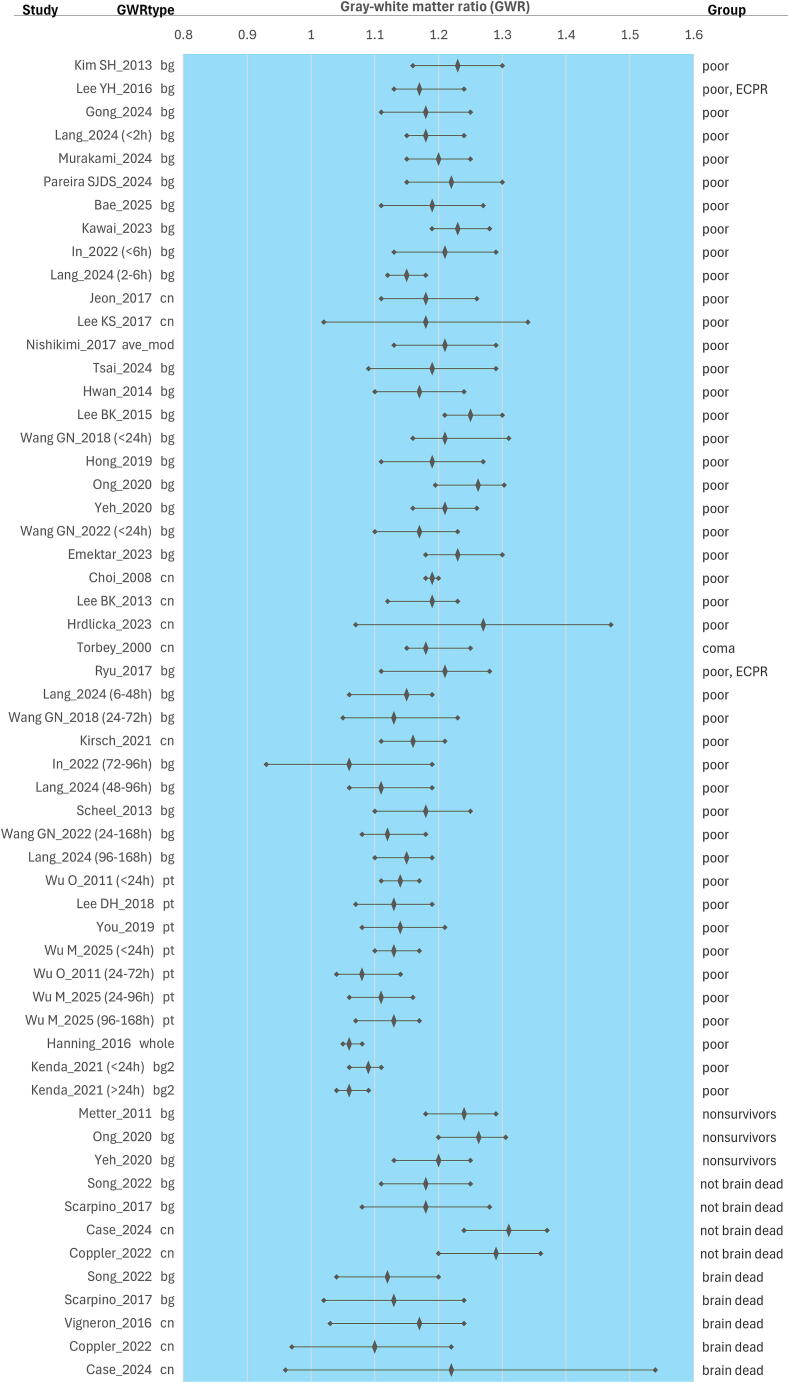
**GWR distribution in adult cardiac arrest and extracorporeal cardiopulmonary resuscitation (ECPR) studies’ unfavorable groups**. The forest plot includes a diamond shape and a line to indicate the median gray-white matter ratio (GWR) with interquartile range or the mean and standard deviation for the unfavorable groups: poor outcome, nonsurvivors, not brain dead, and brain dead, over a blue background. On the left axis, the first author's last name and publication year indicate a study; if studies reported more than one group based on CT acquisition time are indicated in parenthesis for each strata: <2 h, <6 h, 2–6 h, <24 h, >24 h, 6–48 h, 24–72 h, 24–96 h, 72–96 h, 48–96 h, 24–168 h, 96–168 h. The GWR type is shown with the abbreviations ‘bg’,‘cn’, ‘pt’, ‘ave_mod’, ‘whole’, ‘bg2′, referring to the equations used to calculate the GWR: ‘bg’ with basal ganglia (GWR_bg), ‘cn’ with caudate nucleus (GWR_si(CN)), ‘pt’ with putamen (GWR_si(PT)), ‘ave_mod’ with thalamus and cortical gray matter (GWR_ave_mod), ‘whole’ with whole brain gray mat ter (GWR_whole), ‘bg2′ with basal ganglia plus thalamus (GWR_bg2). Equations are included in Table 2 in the Supplemental Materials. On the right axis, study results are displayed by unfavorable outcome group: poor outcome, nonsurvivors; not brain dead, brain dead; groups including ECPR are noted. Studies are ordered from top to bottom, from early to later CT acquisition time, in each outcome group. (For interpretation of the references to color in this figure legend, the reader is referred to the web version of this article.)

## Results

The study selection is summarized in a PRISMA flow diagram (Fig. 1). Five pediatric^4^, ^14^, ^15^, ^16^, ^17^ and 40 adult cardiac arrest studies that reported GWR were included and summarized in Table 1.^1^, ^2^, ^3^, ^6^, ^8^, ^12^, ^13^, ^18^, ^19^, ^20^, ^21^, ^22^, ^23^, ^24^, ^25^, ^26^, ^27^, ^28^, ^29^, ^30^, ^31^, ^32^, ^33^, ^34^, ^35^, ^36^, ^37^, ^38^, ^39^, ^40^, ^41^, ^42^, ^43^, ^44^, ^45^, ^46^, ^47^, ^48^, ^49^, ^50^, ^51^ The average age in pediatric studies ranged from 0.33 to 5.5 years (y). The average age in adult studies ranged from 47 to 71.5 y. One pediatric study reported controls, whose median age was 10.5 y.^16^

**Table 1:** Summary of studies in pediatric and adult cardiac arrest and extracorporeal cardiopulmonary resuscitation (ECPR) reporting gray-white matter ratio (GWR) on head computed tomography (CT).

					**Outcome**				**GWR equations**						**CT timing inclusion criteria**						**Median time from ROSC/cannulation to CT**
**Author (year)**	**Number of patients**	**IHCA or OHCA**	**CPR, ECPR**	**Age (y)**	**Survival**	**Good vs poor**	**Brain death**	**Poor outcome (time assessed)**	**GWR_bg**	**GWR_si(CN)**	**GWR_si(PT)**	**GWR_cortical**	**GWR_average**	**Others**	**≤3 h**	**≤6 h**	**≤12 h**	**≤24 h**	**≤48 h**	**≥48 h**	
***Pediatric***																					
Starling (2015)	78	OHCA	CPR	2.3	●	●		PCPC4–6	●						●	●	●	●			3.3 h
Yang (2019a)	64	OHCA	CPR	4.1		●		PCPC4–6	●	●	●	●	●		●	●	●	●			1.9 h
Yang (2019b)	21	OHCA	CPR	0.8,5		●		PCPC4–6	●						●	●	●	●			−
Lee Y (2022)	56	−	CPR ECPR	5.5	●			PCPC	●	●	●	●	●		●	●	●	●			3 h
Tetsuhara (2021)	70	OHCA	CPR	0.33	●			PCPC			●				●	●	●	●			−
***Adult***																					
Metter (2011)	240	OHCA	CPR	60	●			CPC, mRS	●			●	●		●	●	●	●			4.2 h
Scheel (2013)	98	OHCA IHCA	CPR	61		●		CPC3–5	●			●	●		●	●	●	●	●	●	5 h
Kim SH (2013)	51	OHCA	CPR	53		●		CPC3–5	●			●	●		●						16 min
Hwan Kim (2014)	91	OHCA IHCA	CPR	59		●		CPC3–5	●	●	●				●	●	●	●			55 min
Lee BK (2015)	283	OHCA	CPR	56		●		CPC3–5	●	●	●	●	●		●	●	●	●			50 min
Lee YH (2016)	30	OHCA	ECPR	49		●		CPC3–5	●		●	●	●		●						−
Ryu (2017)	42	IHCA	ECPR	47		●		CPC3–5	●		●	●	●		●	●	●	●	●		−
Wang GN (2018)	58	OHCA IHCA	CPR	53		●		CPC3–5	●		●	●	●		●	●	●	●	●	●	−
Hong (2019)	512	OHCA	CPR	57		●		CPC3–5 (6 m)	●			●	●		●						−
Ong (2020)	79	IHCA	CPR ECPR	66	●	●		CPC3–5	●		●	●	●		●	●	●	●			−
Yeh (2020)	228	OHCA	CPR ECPR	66	●	●		CPC3–5	●	●	●	●	●	●	●	●	●	●			−
In (2022)	78	OHCA	CPR	57		●		CPC3–5 (6 m)	●						●	●				●	−
Wang GN (2022)	94	OHCA	CPR	51		●		CPC3–5	●		●				●	●	●	●	●	●	−
Emektar (2023)	160	OHCA	CPR	71.5		●		CPC3–5	●			●			●	●	●	●			120 min
Kawai (2023)	321	OHCA	CPR ECPR	69		●		CPC3–5 (1 m)	●			●	●		●						79 min
Gong (2024)	166	OHCA	CPR	55		●		CPC3–5 (6 m)	●						●						20 min
Lang (2024)	341	OHCA	CPR	68		●		mRS4–6	●		●				●	●	●	●	●	●	3 h, 84 h
Murakami (2024)	377	OHCA	CPR	69		●		CPC3–5 (1 m)	●			●	●		●						−
Pereira SJDS (2024)	354	OHCA	CPR	59		●		CPC3–5 (6 m)	●						●						0.9 h
Tsai (2024)	443	OHCA	CPR ECPR	62		●		CPC3–5	●		●				●	●	●				103 min
Bae (2025)	51	OHCA	CPR	53		●		CPC3–5	●	●	●				●						−
Scarpino (2017)	160	OHCA IHCA	CPR ECPR	67	●		●	BD, non-BD	●						●	●	●	●			−
Song (2022)	96	OHCA	CPR ECMO	61			●	BD, non-BD	●						●						17 min
Kenda (2021)	262	OHCA	CPR	62		●		CPC3–5	●		●	●			●	●	●	●	●	●	3 h, 94 h
Torbey (2000)	25	−	CPR	64.5		●♦		coma		●					●	●	●	●	●		−
Choi (2008)	28	OHCA	CPR	54		●♦		GOS1–2		●					●	●	●	●			3.9 h
Lee BK (2013)	186	OHCA IHCA	CPR	58		●		CPC3–5		●	●				●	●	●	●			47.5 min
Lee KS (2017)	67	OHCA	CPR	56		●		CPC3–5		●		●			●	●					125 min
Jeon (2017)	39	OHCA	CPR	52		●		CPC3–5 (6 m)		●	●			●	●	●					90 min
Kirsch (2021)	91	OHCA IHCA	CPR ECPR	64		●♦		CPC4–5		●					●	●	●	●	●	●	−
Hrdlicka (2023)	45	OHCA	CPR ECPR	57		●		CPC3–5 (<6m)		●	●	●		●	●	●	●	●	●		4.3 h
Vigneron (2016)	15	−	CPR	51			●♦	BD		●					●	●	●	●			2.95 h
Coppler (2022)	1569	OHCA	CPR	59	●		●	BD, non-BD		●					●	●	●	●			4.2 h
Case (2024)	1133	OHCA	CPR	58	●		●	BD, non-BD		●					●	●	●	●			4.2 h
Wu O (2011)	151	OHCA IHCA	CPR	59		●		mRS4–6			●				●	●	●	●	●	●	−
Lee DH (2018)	258	OHCA IHCA	CPR	63		●		CPC3–5 (6 m)			●				●	●	●	●			86 min
You (2019)	251	OHCA IHCA	CPR	57		●		CPC3–5 (6 m)			●				●	●					70 min
Wu M (2025)	123	−	CPR	57		●		CPC3–5 (3 m)			●				●	●	●	●	●	●	12.4, 62.3, 134.3 min
Hanning (2016)	84	−	CPR	62		●		CPC3–5						●	●	●	●	●			8.4 h
Nishikimi (2017)	77	−	CPR	61		●		CPC3–5						●	●	●					−

**Abbreviations:** OHCA, out-of-hospital cardiac arrest; IHCA, in-hospital cardiac arrest; CPR, cardiopulmonary resuscitation; ECPR, extracorporeal cardiopulmonary resuscitation; ECMO, extracorporeal membrane oxygenation; PCPC, pediatric cerebral performance category; CPC, cerebral performance category; mRS, modified Rankin Score; GOS, Glasgow outcome scale; BD, brain death; ROSC, return of spontaneous circulation. “–” indicates information not found in the publication. Age: Median or mean age in years; calculated with the age of each outcome group if the report had no information on the total cohort. Outcomes: assessed in adults with CPC or mRS or GOS; in pediatric studies, with PCPC. Dots (●) in the Outcome section indicate the groups for which GWR calculations were selected for the review. Studies reporting controls are shown with diamond shape (♦): Torbey (2000) compared controls with comatose patients, Choi (2008) and Kirsch (2021) compared controls with good/poor outcome, and Vigneron (2016) compared controls with brain dead patients. GWR Equation columns refer to equations detailed in the Supplemental Table 2: GWR_bg, GWR with basal ganglia; GWR_si, GWR with single nucleus (CN: caudate nucleus or PT: putamen); GWR_cortical, GWR with cortical gray matter, GWR_average, average of GWR_bg and GWR_cortical. Dots in the GWR equation section columns represent GWR values selected for Figs. 2–4. Of note, in Kenda 2021, thalamus is added to the basal ganglia (GWR_bg2). Dots in the CT timing inclusion criteria section show each study’s inclusion criteria (≤3 h, ≤6 h, ≤12 h, ≤24 h, ≤48 h, ≥48 h). The last column includes the median time interval from ROSC or cannulation to CT acquisition of the study sample or in each CT timing group (in minutes (min) or hours (h))**.**

### Elapsed time from cardiac arrest and CT acquisition (Table 1)

All pediatric and 33/40 adult studies reported completing the CT within 24 h (h) after the cardiac arrest event. When examining Fig. 2, Fig. 3, there is no definitive trend associated with short or long intervals between cardiac arrest and imaging acquisition time.

### Outcomes

Three pediatric studies reported survival^4^, ^16^, ^17^ and three reported good vs poor neurologic outcomes.^4^, ^14^, ^15^ In adults, six studies reported survival,^2^, ^28^, ^45^, ^46^, ^47^ 34 studies reported good vs poor neurologic outcomes, and five studies reported brain dead status among the nonsurvivors^37^, ^38^, ^45^, ^46^ vs another outcome and one study studied brain dead patients vs controls.^47^

### Regions of interest and GWR equations

When examining which gray matter regions of interest were selected for calculating the GWR, we found that basal ganglia (GWR_bg) was used in four pediatric and in 24 adult studies, including one that calculated basal ganglia with thalamus; caudate nucleus was (GWR_si(CN)) used in 10 adult studies; putamen (GWR_si(PT)) was used in one pediatric and four adult studies; a combination of gray matter regions (GWR_whole and GWR_ave-mod) were used in one adult study (Table 1, Fig. 2, Fig. 3, and Fig. 5).

### *GWR and outcome* groups (Fig. 2, Fig. 3)

In pediatric studies, using basal ganglia in GWR (GWR_bg)^4^, ^14^, ^15^, ^16^ or putamen (GWR_si(PT)),^17^ GWR values range from 1.1 to 1.2 across survivors and good-outcome groups, and GWR range 0.96–1.14 across the nonsurvivors and poor-outcome group. In adult studies, GWR using basal ganglia (GWR_bg),^2^, ^7^, ^8^, ^18^, ^19^, ^20^, ^21^, ^24^, ^25^, ^28^, ^31^, ^32^, ^34^, ^36^ caudate nucleus (GWR_si(CN))^1^, ^6^, ^39^, ^40^, ^41^, ^42^, ^43^, ^45^, ^46^, ^47^ and combining regions (GWR_ave-mod)^12^ range from 1.18 to 1.35 across the survivors and good-outcome group and 1.06–1.31 across the unfavorable outcome groups (nonsurvivors, coma, brain dead, poor-outcome). In adult studies, with GWR using putamen (GWR_si(PT)),^48^, ^49^, ^50^, ^51^ using basal ganglia with thalamus (GWR_bg2),^13^ and a combination of gray matter regions (GWR_whole)^3^ ranged 1.1–1.2 across the good-outcome group and 1.06–1.14 in the poor-outcome group. Adult ECPR studies reported values similar to those in cardiac arrest populations without ECPR.^7^, ^8^ In pediatric controls, GWR with basal ganglia (GWR_bg) median was 1.27 in children (median age 10.5 y).^16^ In adult controls, GWR with caudate nucleus (GWR_si(CN)), median ranged from 1.32 to 1.45.^1^, ^39^, ^41^, ^47^

## Discussion

In this review of GWR in pediatric and adult head CT in cardiac arrest studies, GWR ranges overlapped across good- and poor-outcome groups, and across good-outcome and control groups. In general, GWR values were the lowest in the poor-outcome groups, followed by the good-outcome groups, and highest among controls. GWR ranges in pediatrics, with good outcomes, overlapped with adults with poor outcomes. Those differences may be associated with biological age (e.g., myelination) and/or with age-adjusted technical acquisition protocols. We can speculate that differences may also be associated with differences in the causes of arrests between pediatrics and adults.^52^ In both pediatric and adult studies, the greatest separation was observed between the poor-outcome groups and the control groups.

A wide GWR range (1.14–1.56) was recently reported in an adult reference population. That study concluded that a GWR below 1.10 is unlikely in elderly patients without cardiac arrest. However, other studies have suggested higher cutoff values to predict poor neurological outcomes in adult cardiac arrest patients, ranging approximately from 1.10 to 1.24.^53^ Others using adult studies propose cutoff values of GWR from 1.1 to 1.24 to predict poor neurological outcome^2^, ^3^, ^18^, ^19^, ^21^, ^23^, ^39^, ^40^, ^54^; however, the review found that these values are reported in many studies’ good-outcome groups. In adult studies, GWR ranges were lower in the poor-outcome group than in the good-outcome group, but what is apparent is that the ranges across good and poor outcome groups overlap. The ranges of GWR values in patients who ultimately evolved to being classified as brain death were lower than in groups who were not diagnosed as brain death, but the range was very broad.^41^, ^45^, ^46^, ^47^

Among GWR equations using different regions of interest (see Supplementary material Table 2), GWR_bg2 and GWR_whole, and GWR_si(PT) yielded lower ratios than GWR_bg, GWR_si(CN), and GWR_ave-mod in the good-outcome group (see Fig. 3). The spread of GWR ranges is wider and more variable in the unfavorable outcomes (see Fig. 4).

Several studies reported that the elapsed time interval between cardiac arrest and head CT acquisition may alter the range of GWR.^13^, ^24^, ^25^, ^32^, ^50^ We were unable to show clear trends between earlier and later CT imaging; however, our ability to analyze this data was limited by the broad intervals reported across manuscripts. A recent study in adults conducted across three sites, they found a decrease in GWR after the first 24 h in the poor outcome group whereas it did not change in the good outcome group.^49^

We did not find a difference in GWR ranges between ECPR populations and cardiac arrest studies without ECPR.

## Limitations

We excluded adult studies with overlapping datasets and studies where we were unable to extract the information required for this review. Outcome measures used may be a source of heterogeneity; these varied from “poor outcome vs coma”, good outcome as Cerebral Performance Category (CPC) score 1–2 or CPC 1–3, or other scales such as the modified Ranking Scale. We were unable to consider the impact of targeted temperature management that may have been delivered in some studies, given the incomplete reporting of this feature. Lastly, while there should be minimal inter-scanner variability in HU in CT compared to that found in MRI devices, scanners and protocols used to acquire images may be a source of unmeasured difference between studies^55^ and were not reported in the great majority of studies reviewed. Only 10 of the 40 adult cardiac arrest studies report the kilovoltage power (kVp) information, with eight studies reporting 120 kVp, one 100 kVp and another 80 kVp.

## Conclusion

Our review identified different ranges of GWR between pediatric and adult patients following cardiac arrest. GWR in young children was generally lower than in older children and adults, both after cardiac arrest and in controls. The range of GWR in pediatrics with a good outcome overlaps with the range in adults with a poor outcome. Our review suggests the importance of considering age when using GWR after cardiac arrest. Neuroimaging datasets need to be enriched with pediatric participants and pediatric controls to pursue further development and validation of GWR equations as biomarkers in pediatric cardiac arrest studies. Technically, the review suggests that the range of GWR also varies based on the regions of interest included in the equations; readers should be aware of this source of variability when contrasting studies using GWR as a neuroimaging biomarker of hypoxic ischemic injury.^48^, ^50^ Further work is needed to explore sources of variability and reduce the imprecision of GWR as a tool in cardiac arrest studies.

## CRediT authorship contribution statement

**Kie Honjo:** Writing – review & editing, Writing – original draft, Visualization, Validation, Software, Methodology, Formal analysis, Data curation. **Geraldine Goco:** Writing – review & editing. **Ahmed Al Khalifah:** Writing – review & editing. **Nicole McKinnon:** Writing – review & editing. **Barnaby R. Scholefield:** Writing – review & editing, Visualization. **Suzanne Laughlin:** Writing – review & editing, Visualization, Conceptualization. **Anne-Marie Guerguerian:** Writing – review & editing, Writing – original draft, Visualization, Validation, Supervision, Software, Resources, Project administration, Methodology, Investigation, Formal analysis, Data curation, Conceptualization.

## Declaration of competing interest

The authors declare that they have no known competing financial interests or personal relationships that could have appeared to influence the work reported in this paper.

## Appendix A Supplementary material

Supplementary Data 1 Supplementary material Table 2: Gray-white matter ratio (GWR) equations used in cited references and plotted in Figures 2, 3, and 4. Figure 5: Example of head CT axial images indicating placement of circular regions of interest used in GWR equations.
