# Supplementary material for: Gray-white matter ratio in pediatric and adult cardiopulmonary arrest studies: a rapid review
Source: Resusc Plus. 2026 Apr 11;29:101321. doi: 10.1016/j.resplu.2026.101321 (PMC13129448; doi:10.1016/j.resplu.2026.101321)

**Supplementary material:**

**Table 2.** Gray-white matter ratio (GWR) equations used in cited references and plotted in Figures 2, 3, and 4.

| **GWR** | **Equation** |
| --- | --- |
| GWR_bg​ | (CN+PT)/ (CC+PLIC)​ |
| GWR_si(CN) | CN/PLIC​ |
| GWR_si(PT) | PT/PLIC |
| GWR_cortical | (MC1+MC2) / (MWM1+MWM2)​ |
| GWR_ave​rage | (GWR_bg + GWR_cortical) /2 |
| Others: |  |
| GWR_bg​2 | (CN+PT+TM)/ (CC+PLIC)​ |
| GWR_ave​_mod | (TM/PLIC + GWR_cortical) /2 |
| GWR_whole | mean-GM/WM from whole brain |

GWR_bg, GWR basal ganglia; CN, caudate nucleus; PT, putamen; CC, corpus callosum; PLIC, posterior limb of the internal capsule; TM, thalamus; GWR_si, GWR single deep nucleus; GWR_cortical, GWR calculated with cortical gray matter (GM); MC, medial cortex; MWM, medial white matter; MC1&2 and MWM1&2, 1 and 2 indicate two different level of axial slices (1-level at centrum semiovale, 2-level at high convexity). See Figure 5 (B) & (C): GWR_ave_mod, abbreviation of GWR_average_modified, GWR calculated with thalamus and cortical GM.

**Figure 5:** Example of head CT axial images indicating placement of circular regions of interest used in GWR equations.

Circular regions of interest colours: green for gray matter, orange for white matter, placed bilaterally.

(A) basal ganglia in the genu corpus callosum (CC), in the head of caudate nucleus (CN), in the putamen (PT), in the posterior limb of the internal capsule (PLIC), and in the thalamus (TM);

(B) centrum semiovale level in the medial cortex (MC1) and medial white matter (MWM1);

(C) high convexity level in the medial cortex (MC2) and medial white matter (MWM2).

GWR was calculated using the average Hounsfield Unit (HU) from the same region’s bilateral region of interest.


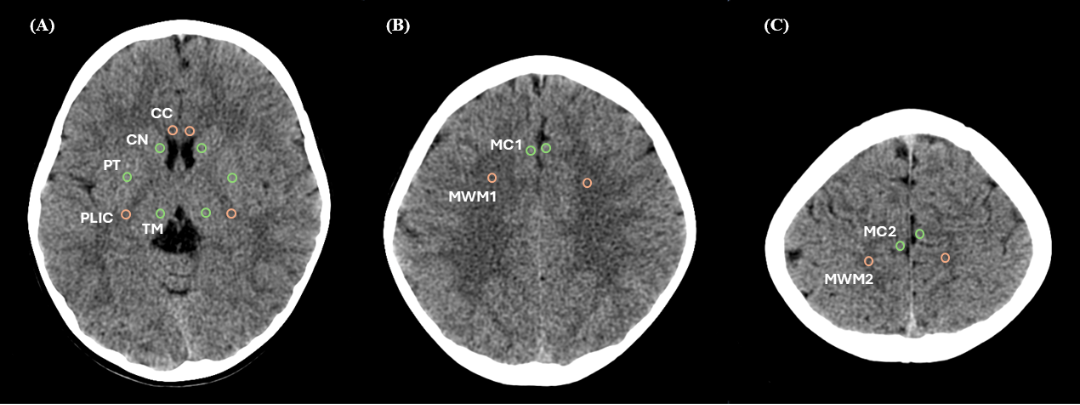

Supplement: Supplementary Data 1 — Supplementary material Table 2: Gray-white matter ratio (GWR) equations used in cited references and plotted in Figures 2, 3, and 4. Figure 5: Example of head CT axial images indicating placement of circular regions of interest used in GWR equations. [file mmc1.docx]
